# Early Life History and Recruitment Processes of a Tropical Anguillid Eel *Anguilla marmorata* to the Pacific Coast, as Revealed by Otolith Sr:Ca Ratios and Microstructure

**DOI:** 10.3390/biology11060803

**Published:** 2022-05-24

**Authors:** Takaomi Arai

**Affiliations:** Environmental and Life Sciences Programme, Faculty of Science, Universiti Brunei Darussalam, Jalan Tungku Link, Gadong, Bandar Seri Begawan BE 1410, Brunei Darussalam; takaomi.arai@ubd.edu.bn; Tel.: +673-2463001

**Keywords:** biogeography, dispersal, freshwater eels, migration, oceanic current, otolith Sr:Ca ratios

## Abstract

**Simple Summary:**

Examination of strontium:calcium (Sr:Ca) ratios in otolith has elucidated substantial information on the life history of fishes. This study has found that a drastic decline in otolith Sr:Ca ratios, indicated the initiation of metamorphosis from larva to juvenile in a tropical anguillid eel *Anguilla marmorata*. Based on the criterion, the current study revealed the early life history and recruitment processes of *A. marmorata*. Furthermore, the larval transportation, dispersion processes and recruitment dynamics to the Pacific coast of *A. marmorata* are determined by means of the otolith microchemical analysis in combination with abiotic parameters such as oceanic currents.

**Abstract:**

Recent progress in otolith microchemistry especially in strontium:calcium (Sr:Ca) ratios has revealed significant features of life histories in fishes. A catadromous eel, *Anguilla marmorata,* has the widest distribution among anguillid eels throughout the Indo-Pacific region. However, its dispersal and recruitment mechanisms in the ocean are still unknown. The temporal and spatial variations of early life history characteristics in a tropical anguillid eel *A. marmorata* were examined by means of otolith Sr:Ca ratios and microstructure to understand the larval transport and recruitment processes to the coasts in the Pacific region. Durations of the larval stage and age at recruitment to the southern part of Japan ranged from 79 to 157 d and 113 to 192, respectively. No significant differences were found between recruitment months in those parameters. The early life characteristics such as larval duration and age at recruitment were constant throughout the recruitment period in the southern part of Japan. The early life history characteristics in combination with the oceanic current regime possibly determine the larval transportation and dispersion processes and further recruitment dynamics to the Pacific coast of *A. marmorata*. The present study also provides useful information on its biogeographic distribution in the species as determined by otolith Sr:Ca ratios and microstructure.

## 1. Introduction

The giant mottled eel *Anguilla marmorata* is widely distributed in the Indo-Pacific region and is one of the most widespread freshwater eels in the region [1,2]. *A. marmorata* has several populations throughout the distribution range and grows to much larger sizes than other eels [3,4,5,6]. Similar to other anguillid eels, this tropical species spawns in the ocean. These larvae are then transported by oceanic currents during the larval stage, before experiencing a distinct metamorphosis into glass eels before recruitment to coastal waters [1,7]. The oceanic life characteristics such as the larval (leptocephalus) phase and metamorphosis timing together with oceanic current systems would determine the biogeographical distribution of anguillid eels. The prospective for prolonged larval transportation in combination with oceanic current systems could be responsible for the worldwide distribution and speciation of the species [8]. However, there is currently little information and evidence available on the spawning areas and larval transportation mechanisms of *A. marmorata.*

Fish otolith is a biomineralized aragonite crystalline structure principally constructed from calcium carbonate with small amount of an organic matrix [9]. Alkaline earth metals and other elements (e.g., Mn, Zn, Pb) likely substitute for calcium (Ca) due to the fact that they have Ca-like properties [9,10,11,12]. These elements are incorporated into otolith growth increments and are affected by environmental factors such as temperature and salinity and physiological factors [9,10,11,12,13]. Furthermore, otolith growth increments are deposited daily in many fish during their larval and juvenile stages [9]. Otoliths are metabolically inert, and hence once the elements are deposited in the otolith, it perpetually records the environmental surroundings encountered by the fish throughout their life history [9,14]. Among elements, strontium (Sr) is widely used to reconstruct fish migration and life history because the Sr concentrations or Sr:Ca ratios in otoliths are positively correlated with salinity in waters [9,10,11,12,13,14].

In the anguillid eels of the genus *Anguilla*, otolith Sr:Ca can reconstruct the life history, migratory history and habitat use throughout their lives [7,8,11]. The application of otolith Sr:Ca ratios to reconstruct the migratory history has revealed a diverse migration strategy in various species such as *A. anguilla* [15,16], *A. japonica* [17,18,19,20,21,22,23,24], *A. rostrata* [25,26], *A. australis schmidtii* and *A. dieffenbachii* [27], *A. marmorata* [28,29,30,31,32], *A. bicolor bicolor* [14,33,34,35] and *A. bicolor pacifica* [31]. Progress in otolith strontoium:calcium (Sr:Ca) ratios techniques in combination with the microstructure has also elucidated many crucial details of the early life histories of anguillid eels such as the timing of onset and length of duration of metamorphosis [36,37,38,39,40,41,42,43,44]. During the early life phase, an abrupt decline in Sr:Ca ratios, coincident with a marked increase in otolith increment widths, indicated the onset of metamorphosis. Metamorphosis is apparently completed before the maximal peak of otolith incremental width. Hence, otolith Sr:Ca ratios are believed to be a powerful tool for reconstructing the life history characteristics in the eels [7,38].

In the present study, life history features such as larval duration and age at recruitment were examined by means of otolith Sr:Ca ratios and microstructure of *A. marmorata* glass eels recruited to the Japanese coast. Temporal variations of early life history characteristics were examined throughout the recruitment period in the southern part of Japan. Furthermore, to understand the spatial variations, previously published data on the early life characteristics of *A. marmorata* in Fiji [44], Indonesia [36,37,39,40], Japan [39,41,43], Philippines [36,39,43] and Taiwan [39,41,43] in the Pacific region were also examined. There have been several studies conducted in an attempt to understand the spatial variation in the early life history of *A. marmorata* [36,37,38,39,40,41,42,43,44]. However, there have been very few studies conducted on the temporal variation throughout the recruitment period in the life history of *A. marmorata* [37] as well as other anguillid eels. It is indispensable to study the spatial and temporal variation of early life history traits for understanding the valid mechanisms of larval transportation and recruitment in the species. The present results establish the foundation for a discussion of larval transportation and recruitment processes to the Pacific coast in relation to the oceanic current systems in the region.

## 2. Materials and Methods

### 2.1. Ethics Statement

Our protocols followed the ethical guidelines for the use of animals of the Universiti Brunei Darussalam (UBD) and were approved by the animal ethics committee at UBD (Approval Code: UBD/RSCH/1.4/FICBF(b)/2021/037; Approval Date: 15 September 2021).

### 2.2. Animals

**Figure 1:**
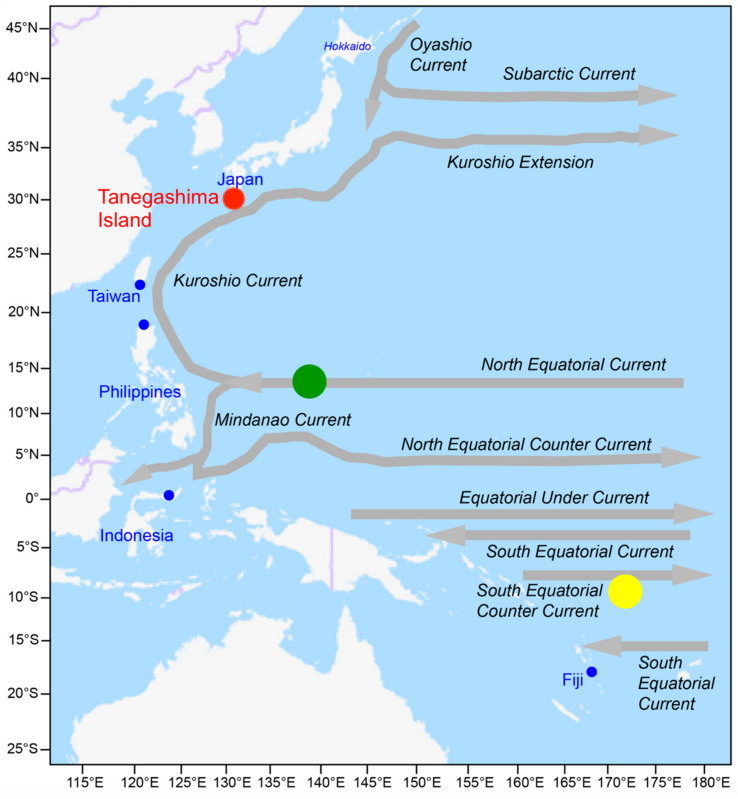
Map showing the Tanegashima Island (red circle) and other sites examined early life history in the previous studies (blue circle), surface currents and a spawning site (green circle) [47,48,49,50] in the North Equatorial Current and an estimated spawning site (yellow circle) [51] in the South Equatorial Current of *Anguilla marmorata* in the Pacific Ocean. The base map was downloaded from the OpenStreetMap (open access) at https://www.openstreetmap.org (accessed on 10 August 2021).

*A. marmorata* glass eels were sampled during the nighttime at the time of the new moon by means of scoop and dip nets on the shore of Tanegashima Island, southern Japan, on 18 December in 1998 and 17 January, 16 February and 17 March in 1999 (Figure 1, Table 1). All specimens were kept for several years in ethanol until ready for experiment. Elemental composition in otolith is not influenced by ethanol preservation [45]. Each total length (TL) was measured and the pigmentation stage was determined following Bertin [46], respectively.

### 2.3. Otolith Preparation

Otoliths were collected from all fish and were fixed in epoxy resin (Struers, Epofix, Copenhagen, Denmark). All otoliths were ground and polished in the sagittal plane by a grinding machine (Struers, Discoplan-TS, Copenhagen, Denmark) and an automated polishing wheel (Struers, Planopol-V, Copenhagen, Denmark) with 6 µm and 1 µm diamond paste, respectively. After polishing, otoliths were cleaned and rinsed using an ultrasonic bath and Milli-Q water for otolith Sr and Ca analyses. 

### 2.4. Otolith Sr and Ca Analyses

All otoliths were platinum and palladium (Pt-Pd) coated for electron microprobe analyses. Sr and Ca levels in otoliths were determined across the longest axis by means of a wavelength-dispersive X-ray electron microprobe (JEOL JXA-8900R, Tokyo, Japan). Calcite (CaCO_3_) and strontianite (SrCO_3_) were used as standards [39,41]. The beam current and accelerating voltage were 12 nA and 15 kv, respectively. The electron beam had a 1 µm diameter while measuring 1 µm space on the otolith. The data depict the mean of three quantifications (4.0 s in each counting time). Microprobe quantification points could be identified along the burning transect on the otolith which were allocated to otolith increments after the otolith increment analysis.

### 2.5. Otolith Increment Analysis

After the Sr and Ca analyses, all otoliths were etched with 0.05 M HCl after removing the coating. Otolith microstructures were observed by means of scanning electron microscope (SEM, Hitachi S-4500, Kyoto, Japan) on the sagittal plane [39,41]. Because otolith increments in *A. marmorata* have validated the daily periodicity [52], similar to other anguillid eels such as *A. japonica* [53,54,55], *A. rostrata* [56] and *A. celebesensis* [57], the increment number can be used as the daily age (Table 1).

### 2.6. Interpretation of Early Life History

**Figure 2:**
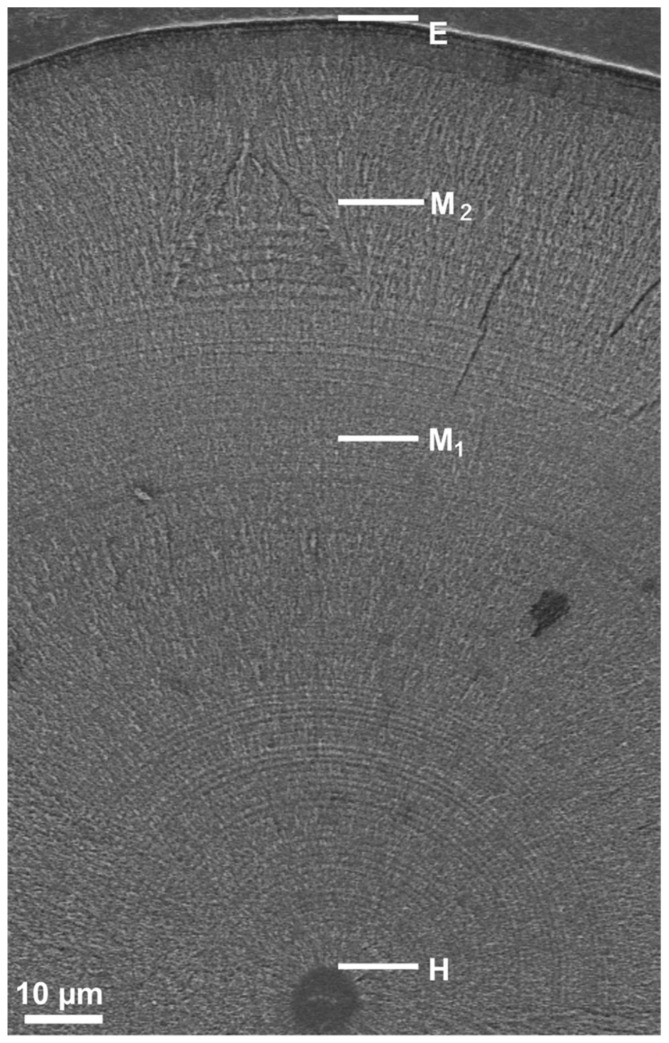
A SEM image shows the otolith microstructure of a *A. marmorata* glass eel collected on the beach on Tanegashima Island, Japan. H, hatch check; M_1_, initiation of metamorphosis; M_2_, completion of metamorphosis; E, otolith edge.

**Figure 3:**
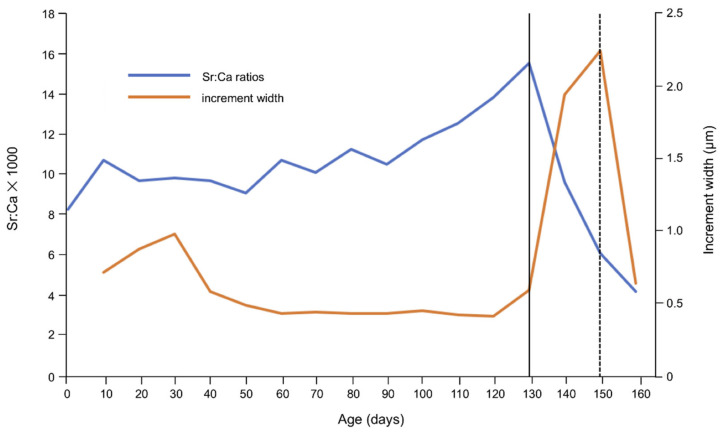
A profile shows fluctuations in otolith Sr:Ca ratios (blue line) and widths of otolith increments (orange line) across the line history transect. The solid and dashed lines suggest age at onset of metamorphosis and age at completion of metamorphosis, respectively.

Early life history characteristics such as the timing of onset of metamorphosis and duration of the leptocephalus stage were determined using SEM photographs (Figure 2) based on previous studies concerning anguillid eels such as temperate species, *A. anguilla* [58], *A. rostrata* [58], *A. japonica* [38,42,59], *A. australis* [60,61] and *A. dieffenbachii* [40] and tropical species, *A. marmorata* [36,37,38,39,40,41,42,43,44,62], *A. celebesensis* [36,37,63], *A. bicolor bicolor* [36], *A. bicolor pacifica* [37,62,64] and *A. reinhardtii* [61]. According to these studies, for otolith Sr:Ca ratios and the microstructure of anguillid eels, the age at which an abrupt increase in the widths of the otolith increments can be seen in combination with a drastic decrease in otolith Sr:Ca ratios is defined as the initiation of metamorphosis (Figure 2 and Figure 3). The length of the metamorphosis phase was determined as the period between the initiation of a significant increase in the widths of the otolith increments and the maximal otolith width (Figure 2 and Figure 3). Age at recruitment was examined by counting the increment number from the presumed hatching check and the edge of the otolith (Figure 2 and Figure 3).

### 2.7. Statistics

Total length, otolith radius and age at the onset of metamorphosis and recruitment among recruitment periods were analyzed by an analysis of variance (ANOVA) to determine if there was significant variation in these parameters between months and thereafter multiple comparison tests were carried out using Scheffe’s test. Significance of the regression slope and correlation coefficient was analyzed by Fisher’s Z-transformation and an analysis of covariance (ANCOVA) which is a model that relies on linear regression wherein the dependent variable is linear to the independent variable.

## 3. Results

The total lengths and the radii of *A. marmorata* glass eels ranged from 44.8 to 54.2 mm and from 133.1 to 163.8 µm, respectively (Table 1). There were no statistically significant differences in total length and otolith radius between months (December 1998, January to March 1999) (ANOVA, *p* > 0.05). Pigmentation stage was determined by either VA or VB, and was limited to only the caudal, rostral and skull parts on the body surface. The pigmentation stage suggests that all glass eels have been newly recruited to the coast.

Durations of larval stage (ages at onset of metamorphosis) ranged from 79 to 157 d (Table 1). There were no statistically significant differences between recruitment months (ANOVA, *p* > 0.05). Lengths of metamorphosis stage ranged from 10 to 27 d (Table 1). No significant difference was found between the four recruitment months (ANOVA, *p* > 0.05). Age at recruitment to the coast ranged from 113 to 192. There were no statistically significant differences in the ages between recruitment months (ANOVA, *p* > 0.05).

**Figure 4:**
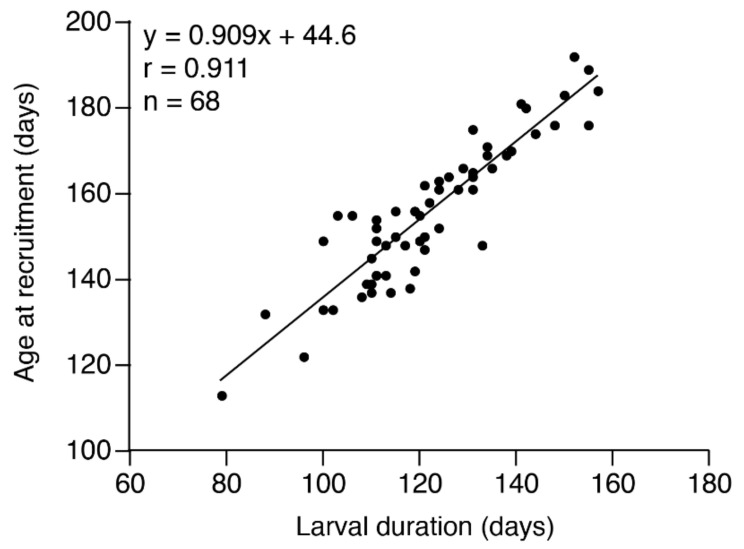
Relationship between larval duration (days) and age at recruitment (days) of *Anguilla marmorata* glass eel recruited to southern Japan.

Linear relationships were found between the duration of the larval (leptocephalus) stage and recruitment (Fisher’s *Z*-transformation, *p* < 0.0001) (Figure 4).

## 4. Discussion

The high Sr:Ca ratios during the larval stage are believed to emanate the substantial accumulation of gelatinous extracellular materials that were incorporated into their bodies just before initiation of metamorphosis [59]. The materials consisted of sulfated glycosaminoglycans (GAG), which are transformed during the metamorphosis [65]. GAG has an affinity to alkaline earth metals especially Sr, and hence, otolith Sr:Ca ratios gradually increased alongside the larval growth. During the metamorphosis stage, GAG is broken down and is excreted outside of the body, leading to the drastic depletion of otolith Sr:Ca ratios [38,59]. In addition, otolith (somatic) growth would accelerate at the initiation of metamorphosis from larva to juvenile [38,59] and hence, otolith growth increments sharply increased as an indication of metamorphosis. After completion of metamorphosis, the growth would gradually decrease to regular growth of the juvenile stage. Otolith Sr:Ca ratios averaged approximately 10 × 10^−3^ at the core, subsequently increased to a maximum level averaging approximately more than 15 × 10^−3^ in the second phase and markedly decreased thereafter toward the edge (Figure 3). These fluctuation patterns in otolith Sr:Ca ratios are commonly found in anguillid eels such as *A. anguilla* and *A. rostrata* [58], *A. australis* [60,61] and *A. dieffenbachii* [40] and tropical eels *A. celebesensis* [36,37,63], *A. marmorata* [36,37,38,39,40,41,42,43,44,62], *A. bicolor bicolor* [36], *A. bicolor pacifica* [37,62,64] and *A. reinhardtii* [61]. Therefore, these otolith features could apply to the early life history analyses in anguillid eels.

The correlation between the duration of the larval age and age at recruitment apparently indicated that juveniles that metamorphosed at a younger age tended to recruit to the coast at a younger age (Figure 4). Metamorphosis could be an important biotic cue to larval transportation, migration and recruitment to coastal waters. The same phenomena were also found in the temperate eels, *A. anguilla* and *A. rostrata* [58], *A. australis* [60,61] and *A. dieffenbachii* [40] and tropical eels *A. celebesensis* [36,37,63], *A. marmorata* [36,37,38,39,40,41,42,43,44,62], *A. bicolor bicolor* [36], *A. bicolor pacifica* [37,62,64], *A. reinhardtii* [61], *A. obscura* and *A. megastoma* [44]. This correlation between the larval duration and the age at recruitment is a common phenomenon and is a key inshore migration mechanism during the oceanic migration phase in anguillid eels.

The life history traits of the larval period, length of metamorphosis stage and recruitment age to the coast were constant throughout four recruitment months from December 1998 to March 1999 in the southern part of Japan (Table 1), possibly as a consequence of constant larval growth during the periods. There are few reports available on the evaluation of temporal variations in the life history characteristics in anguillid eels. In tropical eels, the early life history parameters of *A. marmorata*, *A. celebesensis* and *A. bicolor pacifica*, which recruited to the Indonesian coast, were constant throughout the year [37]. Their stable larval growth and timing of metamorphosis throughout the year would be the result of constant age at recruitment in tropical anguillid eels. Furthermore, monthly spawning with no seasonal spawning was found in tropical anguillid eels [37,66,67], possibly a result of constant life history traits throughout the year. 

The life history traits of *A. marmorata* were essentially the same in the Pacific region, with the mean length of the larval stage ranging from 110 d (Philippines) to 128 d (Indonesia) and the mean recruitment age ranging from 145 d (Philippines, Taiwan and Japan) to 155 d (Indonesia) (Table 2). The larval duration and age at recruitment of *A. marmorata* were found to be constant throughout the year along the Indonesian coast [37]. These findings suggest that early life history traits such as age at recruitment and metamorphosis would be consistent in *A. marmorata* in the Pacific region, regardless of their different geographic locations, oceanic transportation routes and growth histories. However, in Réunion Island, located in the Indian Ocean, mean larval length and mean age at recruitment of *A. marmorata* were 97 and 120 days, respectively [68], shorter than those found in the Pacific region. Differences in the life history traits of *A. marmorata* between the Indian Ocean and the Pacific Ocean may suggest different population structures across the Indo-Pacific.

The mean TLs of *A. marmorata* at recruitment (44.8 to 54.2 mm) were 10 to 20 mm smaller than those of temperate anguillid eels even at the same developmental stage [39]. The smaller sizes of tropical glass eel species, such as *A. bicolor bicolor* (45.5–54.5 mm) [36,68], *A. bicolor pacifica* (48.6–54.5 mm) [37,64], *A*. *celebesensis* (44.7–52.8 mm) [36,37,63], *A. reinhardtii* (47.6–52.2 mm) [61], *A. bengalensis labiata* (51.0–52.5 mm) [68], *A. mossambica* (43.0–53.5 mm) [68,69], *A. obscura* (46.9–53.0 mm) [44] and *A. megastoma* (47.8–53.1 mm) [44], were consistent with the size of *A. marmorata*, when they recruited to tropical coastal areas. Furthermore, differences in TLs of fully developed larvae have also been found between temperate and tropical species. The TLs of fully developed larvae in temperate anguillid eels were found to be 60 mm in *A. japonica* [70], 70 mm in *A. rostrata* [71] and 75 mm in *A. anguilla* [1]. However, those of tropical anguillid eels in the Indo-Pacific region including *A. marmorata* have been found to be approximately 50 mm [62]. The larval growth rate of *A*. *marmorata* was found to be in the range of 0.38 to 0.43 mm day^−1^ [62]. Although the durations of the larval stage were similar between *A. marmorata* and *A. japonica* [43], the growth rate of *A*. *marmorata* is less than that of the temperate eel, *A. japonica* (0.56–0.59 mm day^−1^) [72,73]. The smaller larval growth rate found in *A. marmorata* would be due to the smaller size of the fully developed larval and juvenile at recruitment.

There is little information available on the life history of *A*. *marmorata* throughout the geographical distribution range, although the species is a geographically widespread species in the Indo-Pacific [7,8,74]. Information on the reproduction and migration ecology of *A. marmorata* is still unknown [75], and only one spawning site has been discovered in the North Equatorial Current (NEC) west of the Mariana Islands in the northwest area of the Pacific (Figure 1) [50]. *A. marmorata* larvae entrapped in the Kuroshio Current (KC) would be further transported northward to northern Philippines and East Asian countries. In contrast, the larvae entrapped in the Mindanao Current (MC) would be transported across the east coast of the southern part of the Philippines. Thereafter, other larvae would be further transported westward by means of water flows from the Mindanao Eddy area in the southeast region of the Philippines to the Celebes Sea [47,48] and then *A. marmorata* glass eels recruit to the Sulawesi Island in the east of Indonesia and adjacent to the Celebes Sea areas.

These two transportation routes and mechanisms of *A. marmorata* larvae are supported by the population genetic research [3,4,5,6]. *A. marmorata* specimens from Indonesia, Japan, Philippines and Taiwan were found to belong to the same northern population, which was apparently separated from five other populations dispersed throughout the distribution range. In the northwest Pacific region, *A. marmorata* would migrate downstream to spawn in the NEC and then their larvae would be widely transported and dispersed over 30 degrees of latitude. Differences in the length of the larval stage and the age at recruitment among the sites might be due to the different distances from the spawning site in the NEC, seasonal fluctuations in the velocity of NEC, KC and MC [49] or the fluctuations and dynamics of the coastal currents around the recruitment sites. 

However, in *A. marmorata* from Fiji in the South Pacific, larval transportation mechanisms and routes are highly speculated because little research has been conducted [51]. Further intensive research on the early life history by means of otolith Sr:Ca ratios and the microstructure and geographic distribution of *A. marmorata* larvae in combination with ocean current dynamics and fluctuations is needed to determine the spawning area and larval transportation mechanisms of *A. marmorata* in the South Pacific Ocean.

## 5. Conclusions

The temporal and spatial variations in early life history characteristics in a tropical anguillid eel, *A. marmorata,* in the Pacific region were revealed using otolith Sr:Ca ratios in combination with the microstructure. The life history traits of the larval period, length of metamorphosis stage and recruitment age to the coast were constant throughout the recruitment period. The life history traits of *A. marmorata* were overlapped and essentially the same in the Pacific region, with the mean length of the larval stage ranging from 110 d to 128 d and the mean recruitment age ranging from 145 d to 155 d. Metamorphosis at a younger age caused recruitment to the coast at younger ages, suggesting that metamorphosis is a key factor in determining the inshore migration to coastal areas. The early life history characteristics in combination with the oceanic current regime possibly determine the larval transportation, dispersion processes and recruitment dynamics to the Pacific coast of *A. marmorata*. The present study could provide useful information on its biogeographic distribution in the species based on otolith Sr:Ca ratios and microstructure.

## Figures and Tables

**Table 1:** Early life history characteristics of *Anguilla marmorata* recruited to southern Japan examined in this study.

Month	Number of Specimens	Total Length (mm)		Otolith Radius (µm)		Duration of Larval Stage (Days)		Duration of Metamorphosis Stage (Days)		Age at Recruitment (Days)	
		Mean ± SD	Range	Mean ± SD	Range	Mean ± SD	Range	Mean ± SD	Range	Mean ± SD	Range
December 1998	18	49.9 ± 1.2	48.0–52.1	149.7 ± 8.4	137.1–163.3	120.1 ± 25.5	79–157	15.9 ± 3.2	10–20	150.1 ± 22.9	113–184
January 1999	4	50.7 ± 0.8	49.8–51.5	141.1 ± 6.9	133.1–149.3	122.3 ± 15.5	103–138	21.8 ± 5.7	14–27	161.8 ± 12.4	148–175
February 1999	25	49.2 ± 2.0	44.9–54.2	151.6 ± 6.6	138.6–162.7	123.1 ± 13.4	100–155	17.2 ± 4.0	12–24	154.3 ± 17.0	133–189
March 1999	21	49.1 ± 1.8	44.8–52.0	148.6 ± 6.8	138.2–163.8	123.5 ± 12.8	100–152	20.6 ± 3.4	13–27	160.3 ± 10.7	148–192

**Table 2:** Early life history characteristics of *Anguilla marmorata* recruited to Pacific coast.

Country	Total Length (mm)		Duration of Larval Stage (Days)		Age at Recruitment (Days)		Reference
	Mean	Range	Mean	Range	Mean	Range	
Fiji	51.0–51.5	48.0–56.3	115–113	101–146	152–154	124–183	[44]
Indonesia	50.9	47.9–54.8	120	96–147	152	129–177	[36]
	47.9–52.3		128	114–158	155	144–182	[37]
Japan	49.2–49.7	45.1–54.2	123	100–155	154	133–189	[39,41]
	46.7		118		145		[43]
Philippines	49.9	47.2–51.6	120	105–140	154	136–178	[36]
	49.5		110		145		[43]
Taiwan	50.2–50.3	48.0–53.4	116	92–145	145	116–167	[39,41]
	51.6		112		134		[43]

## Data Availability

Data are provided in the article.
